# A convenient scoring system to distinguish intrahepatic mass-forming cholangiocarcinoma from solitary colorectal liver metastasis based on magnetic resonance imaging features

**DOI:** 10.1007/s00330-023-09873-w

**Published:** 2023-07-01

**Authors:** Keren Shen, Weixing Mo, Xiaojie Wang, Dan Shi, Wei Qian, Jihong Sun, Risheng Yu

**Affiliations:** 1 Department of Radiology, The Second Affiliated Hospital, Zhejiang University School of Medicine, Hangzhou, 310009 China; 2 Department of Radiology, Sir Run Run Shaw Hospital, Zhejiang University School of Medicine, Hangzhou, 310016 China

**Keywords:** Intrahepatic cholangiocarcinoma, Neoplasm Metastasis, Magnetic resonance imaging

## Abstract

**Objectives:**

To develop and validate a diagnostic scoring system to differentiate intrahepatic mass-forming cholangiocarcinoma (IMCC) from solitary colorectal liver metastasis (CRLM).

**Methods:**

A total of 366 patients (263 in the training cohort, 103 in the validation cohort) who underwent MRI examination with pathologically proven either IMCC or CRLM from two centers were included. Twenty-eight MRI features were collected. Univariate analyses and multivariate logistic regression analyses were performed to identify independent predictors for distinguishing IMCC from solitary CRLM. The independent predictors were weighted over based on regression coefficients to build a scoring system. The overall score distribution was divided into three groups to show the diagnostic probability of CRLM.

**Results:**

Six independent predictors, including hepatic capsular retraction, peripheral hepatic enhancement, vessel penetrating the tumor, upper abdominal lymphadenopathy, peripheral washout at the portal venous phase, and rim enhancement at the portal venous phase were included in the system. All predictors were assigned 1 point. At a cutoff of 3 points, AUCs for this score model were 0.948 and 0.903 with sensitivities of 96.5% and 92.0%, specificities of 84.4% and 71.7%, positive predictive values of 87.7% and 75.4%, negative predictive values of 95.4% and 90.5%, and accuracies of 90.9% and 81.6% for the training and validation cohorts, respectively. An increasing trend was shown in the diagnostic probability of CRLM among the three groups based on the score.

**Conclusions:**

The established scoring system is reliable and convenient for distinguishing IMCC from solitary CRLM using six MRI features.

**Clinical relevance statement:**

A reliable and convenient scoring system was developed to differentiate between intrahepatic mass-forming cholangiocarcinoma from solitary colorectal liver metastasis using six MRI features.

**Key Points:**

• *Characteristic MRI features were identified to distinguish intrahepatic mass-forming cholangiocarcinoma (IMCC) from solitary colorectal liver metastasis (CRLM).*

• *A model to distinguish IMCC from solitary CRLM was created based on 6 features, including hepatic capsular retraction, upper abdominal lymphadenopathy, peripheral washout at the portal venous phase, rim enhancement at the portal venous phase, peripheral hepatic enhancement, and vessel penetrating the tumor.*

**Supplementary Information:**

The online version contains supplementary material available at 10.1007/s00330-023-09873-w.

## Introduction

Liver metastasis and intrahepatic cholangiocarcinoma (ICC) are the most common malignant hypoenhancing liver lesions. ICC is the second most common primary malignancy of the liver [1]. On the basis of gross morphologic features, ICC can be classified into three subtypes with the mass-forming type being the most frequent, accounting for 78% of all these lesions [2]. The most common mimic of intrahepatic mass-forming cholangiocarcinoma (IMCC) is liver metastasis, especially that from colorectal cancer [3]. Among the sources of metastatic disease to the liver, colorectal cancers are the most common [4–6]. Approximately half of colorectal liver metastases (CRLMs) first present as a solitary nodule or mass [7]. In the setting of known primary colorectal malignancy, a diagnosis of metastases can be made with confidence. When incidentally encountered, however, the imaging appearance of solitary CRLM is nonspecific and overlaps with IMCC. They are both hypoenhancing lesions with a hyperenhancing rim [3, 8]. In addition, patients with known colorectal cancers could develop IMCC independently of their primary disease. Patients with IMCC or colorectal cancer may share similar clinical features, such as elevated carbohydrate antigen 19–9 (CA19-9) [9]. In general, the differentiation at the histological level is not a problem. However, a small portion of metastatic liver tumors have immunoprofiles similar to those of IMCC [10, 11]. The management strategies for these distinct tumors are divergent, as the only potentially curative treatment for IMCC is surgical resection, while colorectal cancer requires an individual approach with surgical resection plus chemotherapy [12, 13]. Thus, differentiation between IMCC and solitary CRLM could be a true diagnostic challenge to radiologists and clinicians.

Liver magnetic resonance imaging (MRI) is widely used in investigating IMCC and CRLM in clinical practice [14]. Previous studies have attempted to identify useful MRI features to differentiate between IMCC and liver metastasis [3, 15, 16]. However, all these studies included liver metastases of different origins, including colorectal and non-colorectal origins. Metastases originating from different primary malignancies may exhibit different imaging features, thus precluding precise comparisons between these tumors. Moreover, these studies did not provide an easy and simple diagnostic method for hypoenhancing liver lesions based on MRI features.

Therefore, we aimed to identify characteristic MRI features and build a diagnostic scoring system for differentiating IMCC from solitary CRLM.

## Materials and methods

### Study population

**Fig. 1:**
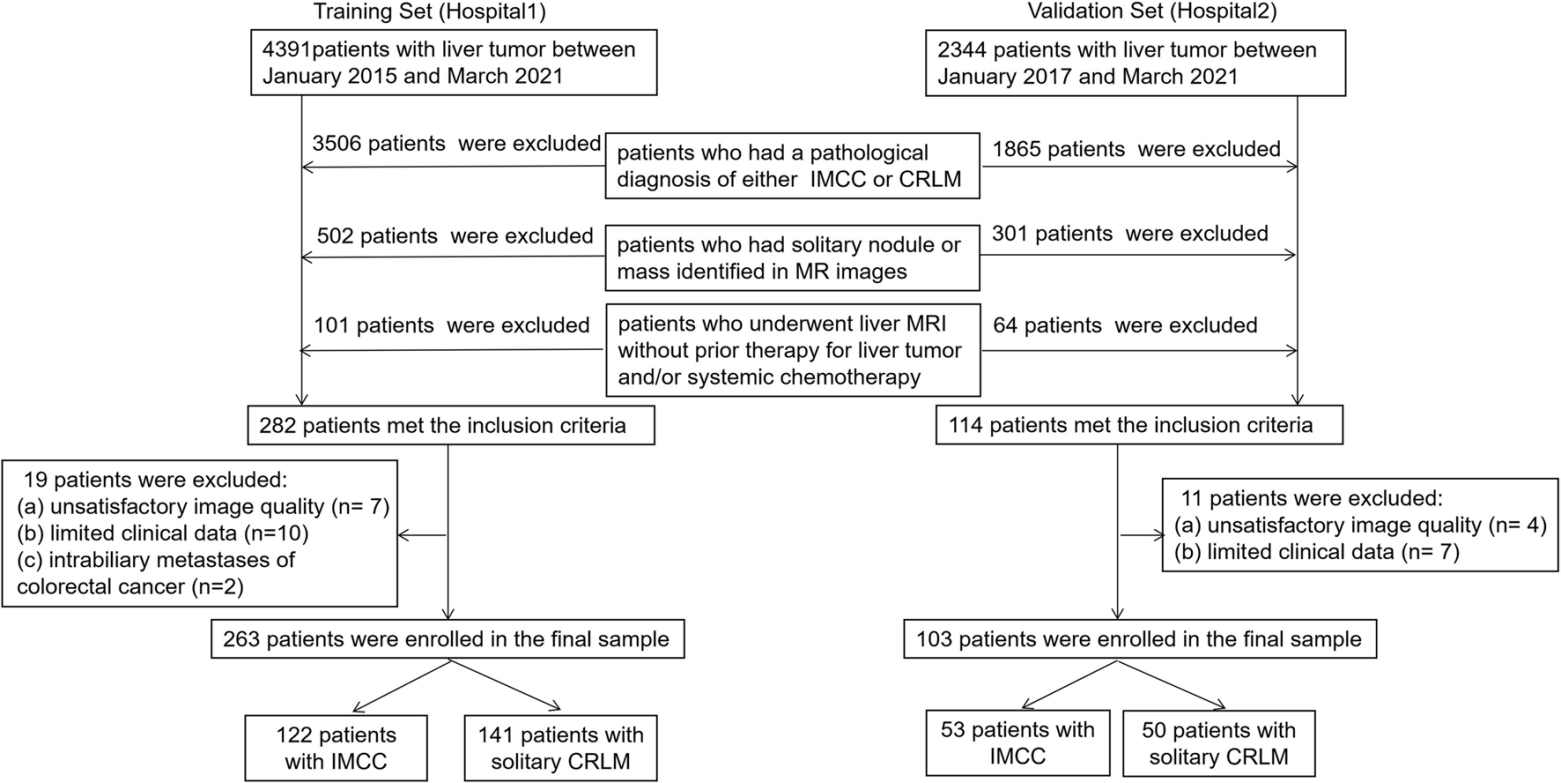
Flowchart of patient selection

We retrospectively included patients consecutively pathologically diagnosed with either IMCC or CRLM at two hospitals. Patients from the Second Affiliate Hospital of Zhejiang University School of Medicine (hospital 1), diagnosed between January 2015 and March 2021, were assigned as the training cohort. Patients from the Sir Run Run Shaw Hospital of Zhejiang University School of Medicine (hospital 2), diagnosed between January 2017 and March 2021, were assigned as the validation cohort. This study was approved by the local ethics committee of the hospital. The requirement for patient informed consent was waived at each hospital. The following inclusion criteria were applied: (a) patients who had a pathological diagnosis of either IMCC or CRLM; (b) patients who had solitary nodule or mass identified in MRI; and (c) patients who underwent liver MRI without prior treatment for liver tumor and/or systemic chemotherapy. The following patients were also excluded: (a) patients with unsatisfactory image quality; (b) patients with limited clinical data; and (c) patients with intrabiliary metastases of colorectal cancer (Fig. 1).

### MRI acquisition

All patients underwent a standardized hepatic MRI protocol on a 3.0-T scanner. The MRI protocol included T2-weighted imaging (T2WI), in- and opposed-phase T1-weighted imaging (T1WI), diffusion-weighted imaging (DWI), and contrast-enhanced T1WI. Extracellular contrast agents were used, including Omniscan (GE Healthcare) and Magnevist (Bayer) at doses of 0.1 and 0.2 mmol/kg, respectively. Detailed MRI parameters varied depending on the clinical protocol at each hospital (Supplemental Table 1).

### Image analysis

Two radiologists (M.W. and S.K. with 13 and 6 years of experience in abdominal imaging, respectively) reviewed the images independently. The interobserver agreement was evaluated. Then, a third experienced abdominal radiologist (Y.R.) with more than 30 years of experience was invited to resolve any disagreements between the two observers. All the reviewers were blinded to pathological results. The cases selected for training and those used for validation were reviewed during the same session. Images were reviewed on a picture archive communication system.

The following qualitative imaging parameters of the lesions were evaluated on the plain scan: (a) shape (round or oval, lobulated or irregular); (b) contour (smooth or nonsmooth margin); (c) homogeneous or heterogeneous intensity on T2WI; (d) homogeneous or heterogeneous intensity on DWI; (e) tumor location; (f) blood products; (g) necrosis; (h) upper abdominal lymphadenopathy; (i) peritumoral bile duct dilatation; (j) hepatic capsular retraction; (k) cirrhosis. Dynamic enhancement characteristics were as follows: (a) dynamic enhancement pattern (progression, fast-in and fast-out, fast-in and slow-out, and others); (b) enhancement type (hyperenhancing or nonhyperenhancing); (c) degree of arterial phase enhancement (none, mild-moderate or strong); (d) arterial phase enhancement pattern (rim enhancement, complete enhancement, partial enhancement); (e) peripheral washout at portal venous phase; (f) rim enhancement at portal venous phase; (g) peripheral hepatic enhancement; (h) dot- or band-like enhancement inside the tumor [17]; (i) vessel penetrating the tumor [18]; (j) vessel encasement; and (k) portal venous thrombosis. Detailed definitions of qualitative parameters were listed (Supplemental Table 2).

For quantitative image analysis, the signal intensity (SI) of the lesions, liver background, and iliopsoas muscle were evaluated. A circular region of interest (ROI) was placed over each entire lesion on enhanced MR images in the precontrast, arterial phase, portal venous phase, and delayed phase. Liver parenchymal intensity was measured using a fixed-sized circular ROI (100 mm^2^) while avoiding major vessels and artifacts. On the basis of these measurements, the lesion-to-liver contrast (LLC) ratio was calculated using the following formula: [(SI_lesion_ − SI_liver_)/SI_muscle_], where SI_lesion_, SI_liver_, and SI_muscle_ are the SIs of the lesion, liver, and iliopsoas muscle on each image. The thickness of arterial phase rim enhancement and the maximal diameter of the lesion were evaluated.

### Clinical data collection

The following characteristics were obtained from the electronic medical records of each patient: age, sex, history of hepatitis B virus (HBV) infection, and tumor markers (alpha-fetoprotein (AFP), carcinoembryonic antigen (CEA) and CA19-9) within 1 week of MRI examination.

### Statistical analysis

Continuous variables were presented as either the mean ± standard deviation (SD) in cases of normal distribution or the median and interquartile range (IQR) for cases with nonnormally distributed data. Categorical data were recorded as frequency (percentage). The clinical data and MRI features between patients with IMCC and patients with solitary CRLM were compared using the Pearson chi-square test, Fisher’s exact test, Student’s test, or the Mann–Whitney *U* test, as appropriate. To evaluate the reproducibility of quantitative features, intraclass correlation coefficient values were calculated. A value > 0.75 was regarded as good agreement. For quantitative features, the interobserver agreement was evaluated by calculating the kappa values, for which > 0.81, 0.61 to 0.80, 0.41 to 0.60, 0.21 to 0.40, and < 0.20 reflected near perfect, substantial, moderate, fair, and slight agreement, respectively [19]. The variables found to have statistical significance in the univariate analysis were subjected to ridge regression analysis to minimize multicollinearity and then incorporated into a logistic regression model to identify independent predictors for differentiating IMCC from solitary CRLM. To derive a simple-to-compute scoring system, regression coefficients were converted to weighted scores by dividing each regression coefficient by the smallest coefficient and rounding to the nearest integer [20]. For each patient, the scores that corresponded to the related variables were added together to generate an overall score. Calibration was assessed using the Hosmer–Lemeshow goodness-of-fit test. The discrimination performance of the model was assessed using the area under the receiver operating curve (AUC), and the optimum cutoff point was chosen for optimal sensitivity and specificity. Considering the higher prevalence of CRLM than IMCC, we adjusted positive predictive value (PPV), negative predictive value (NPV), and accuracy according to the disease prevalence. PPV, NPV, and accuracy were calculated at a CRLM to IMCC ratio of 5:1 and 10:1 [21, 22]. A comparison between the AUCs of different models was performed using the DeLong nonparametric method. *p* values < 0.05 were considered to indicate a significant difference. All statistical analyses were performed by using SPSS 23.0 and MedCalc 19.0.4.

## Results

### Clinical characteristics in patients

Overall, 366 patients were enrolled in this study. A total of 263 patients—122 with IMCC and 141 with solitary CRLM—were enrolled as the training cohort. A total of 103 patients were studied as the validation cohort, which contained 53 IMCC and 50 solitary CRLM patients. The age of IMCC patients was higher than that of solitary CRLM patients in the training cohort (*p* < 0.05), but not in the validation cohort (*p* = 0.183). The HBV infection rate was significantly higher in patients with IMCC than in those with CRLM in both cohorts (both *p* < 0.05). The CA19-9 level of the IMCC group was higher than that of the solitary CRLM group in both cohorts (both *p* < 0.001). There was no significant difference in sex or the levels of AFP and CEA between the IMCC and solitary CRLM groups in either cohort (Table 1).

**Table 1:** Comparison of patients in clinical characteristics

	Training cohort			Validation cohort		
	Patients with IMCC (*n* = 122)	Patients with CRLM (*n* = 141)	*p* value	Patients with IMCC (*n* = 53)	Patients with CRLM (*n* = 50)	*p* value
Gender			0.478			0.317
Male/female	78/44	96/45		31/22	34/16	
Age (years)			0.001*			0.183
Mean (SD)	65 (10)	61 (9)		61.9 (10.6)	63.2 (9.5)	
HBs-Ag			0.001*			0.016*
Presence	25 (20.5%)	8 (5.7%)		14 (26.4%)	4 (8%)	
CA 19–9 (U/mL)			0.001*			0.001*
Median (IQR)	137.2 (11.4–3644.2)	13.8 (4.3–63.2)		393.6 (30.5–926.0)	22.3 (9.1–54.8)	
CEA (ng/mL)			0.693			0.056
Median (IQR)	3.5 (2.2–7.9)	10.0 (3.9–36.0)		5.6 (2.9–18.1)	14.1 (5.2–39.3)	
AFP (ng/mL)			0.172			0.106
Median (IQR)	3.0 (2.2–5.3)	2.9 (2.4–3.9)		3.2 (2.6–4.2)	2.8 (1.9–3.6)	

Abbreviations: *IMCC* intrahepatic mass-forming cholangiocarcinoma, *CRLM* colorectal liver metastasis, *SD* standard deviation, *IQR* interquartile range, *CA 19–9* carbohydrate antigen 19–9, *CEA* carcinoembryonic antigen, *AFP* alpha-fetoprotein

^*^p value < .05

### Imaging features in patients

To determine the most relevant predictors of distinguishing patients with IMCC from those with CRLM, univariate analysis of the MRI features was conducted. Seventeen qualitative imaging variables, including shape (*p* < 0.001), contour (*p* < 0.001), T2WI signal (*p* < 0.001), DWI signal (*p* < 0.001), upper abdominal lymphadenopathy (*p* < 0.001), peritumoral bile duct dilatation (*p* < 0.001), hepatic capsular retraction (*p* < 0.001), cirrhosis (*p* < 0.001), dynamic enhancement pattern (*p* = 0.013), enhancement type (*p* < 0.001), arterial phase enhancement pattern (*p* = 0.002), peripheral washout at portal venous phase (*p* < 0.001), rim enhancement at portal venous phase (*p* < 0.001), dot- or band-like enhancement inside the tumor (*p* = 0.006), peripheral hepatic enhancement (*p* < 0.001), vessel penetrating the tumor (*p* < 0.001), and portal venous thrombosis (*p* < 0.001) were significantly different between IMCC and solitary CRLM patients (Table 2). For quantitative variables, the maximum diameter of IMCC was significantly larger than that of CRLM (*p* < 0.001). Other quantitative imaging parameters did not differ between the two groups (Supplemental Table 3).

**Table 2:** Comparison of the qualitative imaging variables between IMCC and solitary CRLM in the training cohort

	Patients with IMCC (*n* = 122)	Patients with CRLM (*n* = 141)	*p* value
Location			
Left/right/caudate lobe	50/66/5	41/97/3	0.055
Shape			
Round or oval/lobulated/irregular	26/19/77	97^a^/24^b^/20^c^	< 0.001*
Contour			
Smooth/non-smooth	24/98	88/53	< 0.001*
T2WI			
Homogeneous/heterogeneous	38/84	91/50	< 0.001*
DWI			
Homogeneous/heterogeneous	71/51	127/14	< 0.001*
Blood products			
Absence/presence	106/16	126/15	0.535
Necrosis			
Absence/presence	113/0	126/15	0.360
Upper abdominal lymphadenopathy			
Absence/presence	53/69	130/11	< 0.001*
Peritumoral bile duct dilatation			
Absence/presence	74/48	132/9	< 0.001*
Hepatic capsular retraction			
Absence/presence	50/72	130/11	< 0.001*
Cirrhosis			
Absence/presence	102/20	139/2	< 0.001*
Dynamic enhancement pattern			
Progression/fast-in and fast-out/fast-in and slow-out/others	44/6/57/15	72^a^/19^a^/42^b^/8^b^	0.001*
Enhancement type			
Hypoenhancing/hyperenhancing	69/53	109/32	< 0.001*
Degree of arterial phase enhancement			
None/mild-moderate/strong	3/67/52	4^a^^,b^/96^b^/41 ^a^	0.072
Arterial phase enhancement pattern			
Rim enhancement/complete enhancement/partial enhancement	52/31/39	85^a^/35^a^/21^b^	0.002*
Peripheral washout at portal venous phase			
Absence/presence	114/8	118/43	< 0.001*
Rim enhancement at portal venous phase			
Absence/presence	38/84	9/132	< 0.001*
Dot- or band-like enhancement inside the tumor			
Absence/presence	32/90	60/81	0.006*
Peripheral hepatic enhancement			
Absence/presence	50/72	127/14	< 0.001*
Vessel penetrating the tumor			
Absence/presence	38/84	125/16	< 0.001*
Vessel encasement			
Absence/presence	13/109	27/114	0.056
Portal venous thrombosis			
Absence/presence	97/25	138/3	< 0.001*

Abbreviations: *IMCC* intrahepatic mass-forming cholangiocarcinoma, *CRLM* colorectal liver metastasis, *T2WI* T2-weighted imaging, *DWI* diffusion-weighted imaging

^a,b,c^The same letter markers indicated no statistical differences

^*^p value < .05

The interobserver agreement on qualitative imaging variables evaluation by the two radiologists was near perfect or substantial (kappa value: 0.632 to 1.000). The reproducibility of the quantitative features was in good agreement (intraclass correlation coefficient value: 0.804 to 0.966) (Supplemental Tables 4 and 5).

### Development of the primary predictive model

Variables considered significantly different in the univariate analysis were included in the ridge regression analysis to minimize multicollinearity. As presented in the ridge trace curve (Supplemental Fig. 1), when the *K* value was 0.6, the ridge trace presented with the standardized coefficients of variables was stable, and the model was significant (*p* < 0.001). At this point, 11 MRI features showed significant differences between IMCC and solitary CRLM patients (Supplemental Table 6). For further verification, multivariate logistic regression was performed to demonstrate six independent factors: hepatic capsular retraction, upper abdominal lymphadenopathy, peripheral washout at the portal venous phase, rim enhancement at the portal venous phase, peripheral hepatic enhancement, and vessel penetrating the tumor (all *p* < 0.001) (Table 3). These six MR features were adopted to develop the scoring model. The Hosmer–Lemeshow goodness-of-fit test showed good calibration of this primary model (*p* = 0.451), and the AUC of the primary predictive model was 0.954 (95% CI 0.922–0.976; *p* < 0.001).

**Table 3:** Multivariate regression analysis for MRI features and the weighted score of independent predictors

	*B*	*p*	OR	95% CI for OR		Weighted score
				Lower	Upper	
Hepatic capsular retraction (absence)	2.259	< 0.001	9.573	3.297	27.795	1
Upper abdominal lymphadenopathy (absence)	2.504	< 0.001	12.233	3.847	38.903	1
Peripheral washout at the portal venous phase (presence)	2.148	< 0.001	8.571	2.163	33.966	1
Rim enhancement at the portal venous phase (presence)	2.979	< 0.001	19.664	5.620	68.799	1
Peripheral hepatic enhancement (absence)	2.642	< 0.001	14.044	4.999	39.449	1
Vessel penetrating the tumor (absence)	2.050	< 0.001	7.769	3.068	19.673	1

### Development of the scoring system

**Fig. 2:**
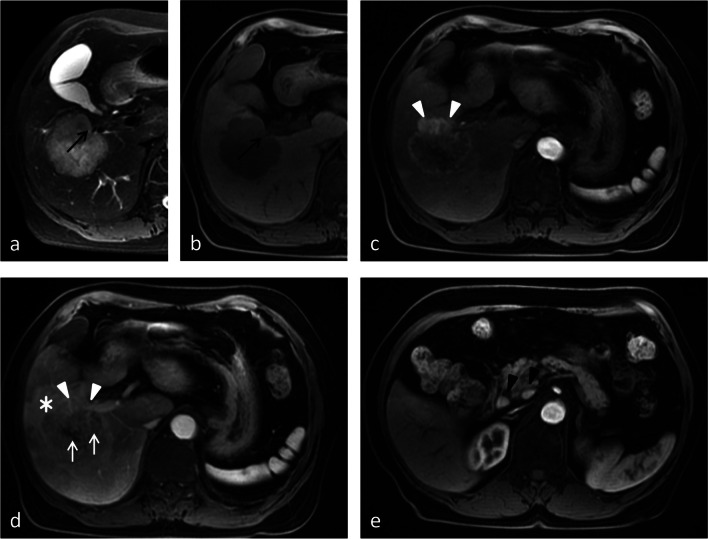
Images from a 70-year-old female with IMCC. **a** The T2-weighted image and (**b**) T1-weighted image show a 5-cm lesion in segment V-VI of the liver with hepatic capsular retraction (black arrow). **c** The arterial phase image shows partial hyperenhancement (white arrowhead). **d** The portal venous phase image shows continuous partial enhancement (white arrowhead), peripheral hepatic enhancement (star), and vessel penetrating the tumor (white arrow). **e** The portal venous phase image at a lower level shows the enlarged lymph nodes around the pancreatic head (black arrowhead). Thus, a score of 0 was assigned for this patient

**Fig. 3:**
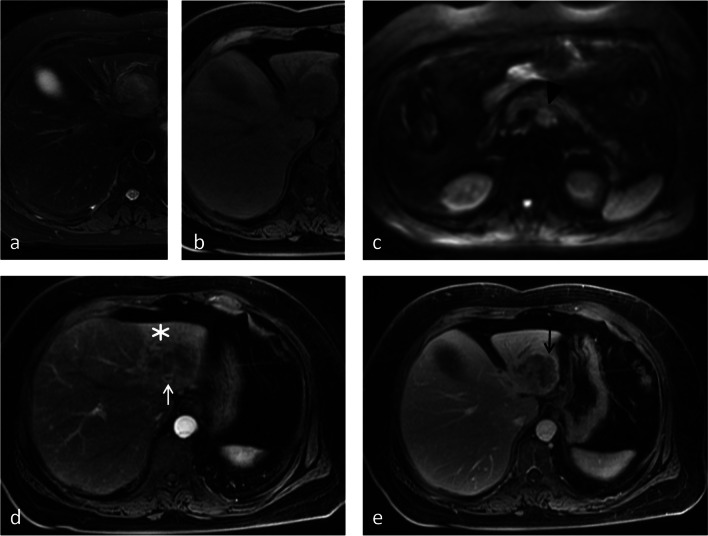
Images from a 78-year-old female with IMCC. **a** The T2-weighted image and (**b**) T1-weighted image show a 4.5-cm lesion in segment II of the liver without hepatic capsular retraction. **c** The DWI image shows an enlarged retroperitoneal lymph node (black arrowhead). **d** The arterial phase image shows peripheral hepatic enhancement (star) and vessel penetrating the tumor (white arrow). **e** The portal venous phase image shows rim enhancement (black arrow). Thus, a score of 2 was assigned for this patient

**Fig. 4:**
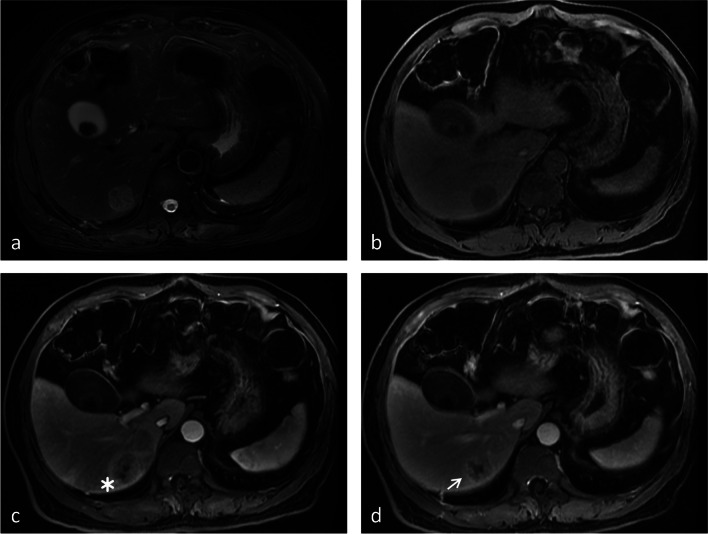
Image from a 76-year-old male with CRLM. **a** The T2-weighted image and (**b**) T1-weighted image show a 2.6-cm lesion in segment VI of the liver. **c** The arterial phase image shows peripheral hepatic enhancement (star). **d** The portal venous phase image shows continuous rim enhancement (white arrow). Hepatic capsular retraction, vessel penetrating the tumor, and upper abdominal lymphadenopathy are not shown. Thus, a score of 4 was assigned for this patient

**Fig. 5:**
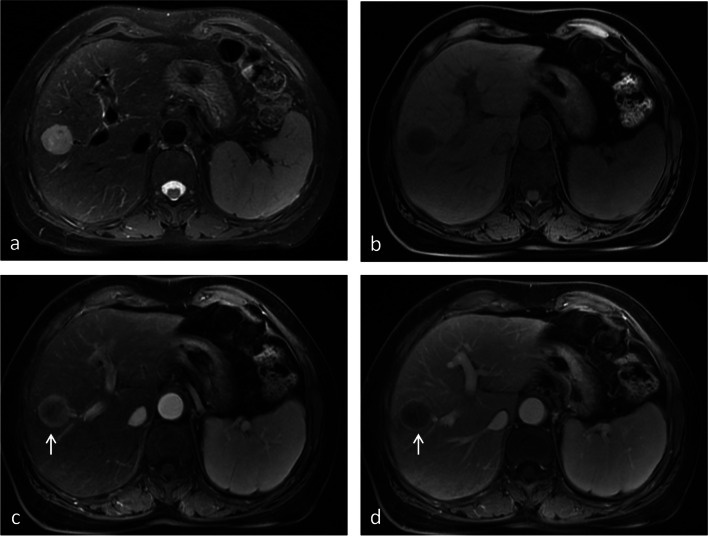
Images from a 66-year-old female with CRLM. **a** The T2-weighted image and (**b**) T1-weighted image show a 2.5-cm lesion in segment VIII of the liver. **c** The arterial phase image shows rim enhancement of the lesion (white arrow). **d** The portal venous phase image shows peripheral washout and continuous rim enhancement (white arrow). Hepatic capsular retraction, peripheral hepatic enhancement, vessel penetrating the tumor, and upper abdominal lymphadenopathy are not shown. Thus, a score of 6 was assigned for this patient

**Fig. 6:**
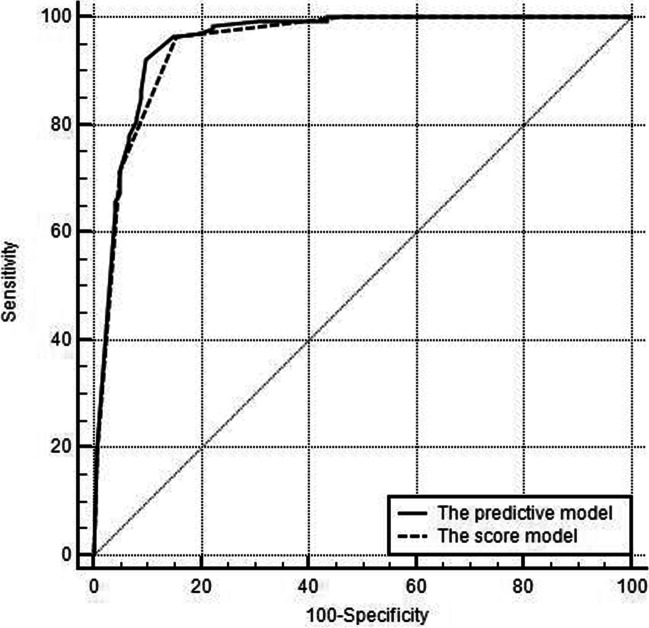
ROC curves of the predictive model and the score model

Weighted scores were assigned to MRI features based on multivariate analysis results to build a scoring system. Coincidentally, the six MRI features were all assigned 1 point (Table 3). The absence of hepatic capsular retraction, upper abdominal lymphadenopathy, peripheral hepatic enhancement, and vessel penetrating the tumor was assigned 1 point each. The presence of peripheral washout at the portal venous phase and rim enhancement at the portal venous phase was assigned 1 point each. For each patient, the individual scores that correspond to the predictors were summed together to produce an overall score ranging from 0 to 6 points (Figs. 2, 3, 4, and 5). The higher the score was, the more likely the lesion was CRLM. The Hosmer–Lemeshow goodness-of-fit test indicated good calibration of this scoring model (*p* = 0.918). The AUC of this distinguishing scoring system, measured by receiver operating characteristic (ROC) curve analysis, was 0.948 (95% CI 0.914–0.971, *p* < 0.001) (Fig. 5). At a cutoff score of 3 points, the performance of the model showed a sensitivity of 96.5%, a specificity of 84.4%, a PPV of 87.7%, a NPV of 95.4%, and an accuracy of 90.9% for distinguishing IMCC from solitary CRLM. After adjusting for disease prevalence, the PPV and accuracy increased to 96.9–98.4% and 94.4–95.4%, respectively. The NPV decreased to 70.4–82.7% (Table 4). The comparison of ROC curves showed no significant difference between the primary predictive model and the score model (*p* = 0.086), indicating that the score model made full use of the value of the primary predictive model (Fig. 6).

**Table 4:** The performance of the scoring system in the training and validation cohorts

	Training cohort	Validation cohort
Sensitivity	0.965 (0.919–0.988)	0.920 (0.808–0.978)
Specificity	0.844 (0.768–0.904)	0.717 (0.577–0.832)
Positive likelihood ratio	6.19 (4.09–9.37)	3.25 (2.10–5.03)
Negative likelihood ratio	0.042 (0.018–0.100)	0.112 (0.043–0.290)
PPV	0.877 (0.825–0.915)	0.754 (0.665–0.826)
PPV (adjust for a CRLM to IMCC ratio of 5:1)	0.969 (0.953–0.979)	0.942 (0.913–0.962)
PPV (adjust for a CRLM to IMCC ratio of 10:1)	0.984 (0.976–0.989)	0.970 (0.955–0.980)
NPV	0.954 (0.897–0.980)	0.905 (0.785–0.961)
NPV (adjust for a CRLM to IMCC ratio of 5:1)	0.827 (0.668–0.912)	0.642 (0.409–0.824)
NPV (adjust for a CRLM to IMCC ratio of 10:1)	0.704 (0.501–0.850)	0.473 (0.257–0.700)
ACC	0.909 (0.867–0.941)	0.816 (0.727–0.885)
ACC (adjust for a CRLM to IMCC ratio of 5:1)	0.944 (0.910–0.969)	0.886 (0.808–0.940)
ACC (adjust for a CRLM to IMCC ratio of 10:1)	0.954 (0.921–0.976)	0.902 (0.827–0.951)

Values in parentheses are 95% confidence intervals

Abbreviations: *PPV* positive predictive value, *NPV* negative predictive value, *ACC* accuracy, *CRLM* colorectal liver metastasis, *IMCC* intrahepatic mass-forming cholangiocarcinoma

To apply this scoring system conveniently in practice, we further divided it into three score ranges as follows: 0–2 points; 3–4 points; and 5–6 points. Among the scoring ranges, the probability of patients with solitary CRLM was 0% for the first (0 to 2 points) range, 44.4% for the second range (3–4 points), and 94.4% for the last range (5–6 points) (Table 5).

**Table 5:** Diagnostic probability of solitary CRLM in different score ranges in the training and validation cohorts

Score range	Training cohort			Validation cohort		
	Number of CRLM	Total number	Diagnostic probability of CRLM	Number of CRLM	Total number	Diagnostic probability of CRLM
0 to 2 points	0	66	0%	1	21	4.8%
3 to 4 points	40	90	44.4%	15	45	33.3%
5 to 6 points	101	107	94.4%	34	36	94.4%

Abbreviations: *CRLM* colorectal liver metastasis

### Validation of the established scoring system

The validation of the scoring system showed satisfactory results. The Hosmer–Lemeshow goodness-of-fit test showed good calibration (*p* = 0.768). The AUC of the scoring system was 0.903 (95% CI 0.829–0.953, *p* < 0.001) in the validation cohort. At a cutoff score of 3 points, the model had a sensitivity, a specificity, a PPV, a NPV, and an accuracy of 92.0%, 71.7%, 75.4%, 90.5%, and 81.6%, respectively. Similar to the training cohort results, the PPV and accuracy increased after adjusting for disease prevalence while the NPV decreased (Table 4). The proportion of patients with solitary CRLM increased with increasing score in the validation cohort (Table 5).

## Discussion

In this study, we developed a convenient scoring system to differentiate between IMCC and solitary CRLM based on commonly used MRI features. The scoring system consisted of six MRI features, which were each assigned 1 point. The overall score ranged from 0 to 6 points: the higher the score was, the more likely the lesion was CRLM. The model showed good performance for distinguishing between the two common hypoenhancing liver lesions.

Previous studies have proposed imaging characteristics for differentiating between IMCC and CRLM. Central necrosis is thought to be specific for CRLM, especially in large lesions. On the other hand, the center of IMCC is characterized by abundant fibrosis, typically in the absence of necrosis [23]. However, in our study, the rate of central necrosis was not significantly different between these tumors, owing to the low rate (10.6%) in CRLM. One possible reason is that the included solitary CRLMs were too small to have necrosis. Target sign on DWI, capsular retraction, segmental biliary dilatation, and heterogeneous intensity on T2WI were suggested to be helpful to differentiate IMCC from liver metastasis in a previous study [15]. These four features also showed significant differences between IMCC and CRLM in our study. However, it should be noted that these variables can only be viewed as associated factors instead of independent risk or protective factors because only univariate analysis was performed. Whether they were truly relevant to IMCC and CRLM required further statistical verification.

To build a convenient scoring system, univariate analysis was performed to obtain the relevant predictors that were significantly different between these tumors. The HBV infection rate and the level of CA19-9 were significantly different between patients with IMCC and those with CRLM. Since the desired scoring system was based on MRI findings, the patient’s clinical parameters were not incorporated into the model. A total of six MRI features were finally selected to build the scoring system: four of these features (hepatic capsular retraction, peripheral hepatic enhancement, vessel penetrating the tumor, and upper abdominal lymphadenopathy) supported the diagnosis of IMCC, and the remaining two features (peripheral washout at the portal venous phase and rim enhancement at the portal venous phase) supported CRLM. The selected MRI features all showed substantial or near perfect interobserver agreement, making the model more robust for clinical practice.

Hepatic capsular retraction was observed in 20–62% of IMCC cases in the literature, which was consistent with our research findings (41%) [18, 24, 25]. IMCC frequently has prominent fibrous stroma, often inducing chronic bile duct obstruction and atrophy of the adjacent liver parenchyma, collectively contributing to retraction of the hepatic capsule [26]. Most metastases that contact the hepatic capsule penetrate the capsule instead of causing capsular retraction [27]. For IMCC, occlusion of the intrahepatic bile duct can also cause peripheral bile duct dilatation and cholangitis. Peripheral hepatic enhancement on MRI may reflect such cholangitis [28]. Vessel penetrating the tumor has been found in IMCC and hepatocellular carcinoma. Intratumoral vessels were defined as discontinuous and tortuous vessels in tumors, which were considered to be related to the microvascular invasion of IMCC [17, 18]. However, the specific mechanism behind this MRI feature remains to be further studied. Upper abdominal lymphadenopathy was another indication for IMCC diagnosis. IMCC patients exhibited a high rate of lymph node metastases at the time of diagnosis. The majority of malignant regional lymph nodes were periportal [29]. Colorectal carcinoma has different lymphatic metastasis routes, with paracolic lymph node metastasis being most common, resulting in less upper abdominal lymphadenopathy [30].

The two MRI features supporting the diagnosis of CRLM were evaluated at the portal venous phase, and both reflected lesion enhancement modes. It was difficult to completely breakdown all the patterns of lesion enhancement. Therefore, we evaluated the overall impression of the whole dynamic enhancement process and the specific morphology of the lesion at each phase. Among several features used to describe enhancement modes, the peripheral washout and rim enhancement at the portal venous phase were the most valuable features for distinguishing IMCC from solitary CRLM. Consistent with previous studies, the most prevalent enhancement pattern of IMCC was progressive and fast-in and slow-out [31]. Thus, peripheral washout at the portal venous phase was uncommon (6.5%) in IMCC in our study. Peripheral rim enhancement has been recognized as one of the characteristic findings of metastatic tumors [32, 33]. However, this feature was also found in 50–100% of IMCCs [34, 35]. In our study, 93.6% of CRLMs and 75% of IMCCs showed rim enhancement at the portal venous phase. Based on our scoring system, more features may need to be analyzed to diagnose a lesion with rim enhancement at portal venous phase as IMCC.

Globally, the prevalence of CRLM is higher than IMCC [21, 22]. However, in our cohorts, the ratio of CRLM to IMCC was almost balanced. One of the reasons was that the incidence of cholangiocarcinoma in our region was much higher than that in North America and Europe, while the incidence of colorectal cancer was lower than that in North America and Europe [36]. Besides, the two hospitals in our study were both tertiary hospitals. The patients always had advanced tumors. Quite a few patients with colorectal cancer had multiple CRLMs. For better clinical applications, we adjusted the results based on disease prevalence. As the CRLM prevalence increased, the PPV increased while the NPV decreased. So our model was especially good for positively identifying CRLM. The diagnosis of IMCC based on our model required caution.

The present study has several limitations that must be acknowledged. First, it was a retrospective study with inherent selection bias. Second, IMCC and CRLM were not subdivided based on histological type. Different histological types of metastases may exhibit different MRI features. Third, the training cohort and validation cohort were evaluated by the same reviewers. The MR scanners and protocols were similar in the two hospitals. An external validation by different radiologists and using different scanners and protocols would better evaluate the model’s reproducibility and generalizability. In addition, the prediction accuracy of the scoring system in the validation cohort was somewhat lower than that in the training cohort, which might be related to biases caused by the relatively small sample size of the validation cohort.

In conclusion, we established and validated an efficient and convenient-to-use scoring system for discriminating IMCC from CRLM based on MRI features. Only the six most meaningful factors were incorporated into this scoring system. The model has potential implications for treatment decision-making.

### Supplementary Information

Below is the link to the electronic supplementary material.

Supplementary file1 (PDF 355 KB)

## Abbreviations

AFP: Alpha-fetoprotein
CA19-9: Carbohydrate antigen 19–9
CEA: Carcinoembryonic antigen
CRLM: Colorectal liver metastasis
HBV: Hepatitis B virus
ICC: Intrahepatic cholangiocarcinoma
IMCC: Intrahepatic mass-forming cholangiocarcinoma
IQR: Interquartile range
LLC: Lesion-to-liver contrast
NPV: Negative predictive value
PPV: Positive predictive value
SD: Standard deviation
SI: Signal intensity

## Declarations

### Guarantor

The scientific guarantor of this publication is Risheng Yu.

### Statistics and biometry

No complex statistical methods were necessary for this paper.

### Methodology

• retrospective

• diagnostic or prognostic study

•multicenter study
