# Supplementary material for: A convenient scoring system to distinguish intrahepatic mass-forming cholangiocarcinoma from solitary colorectal liver metastasis based on magnetic resonance imaging features
Source: Eur Radiol. 2023 Jul 1;33(12):8986–98. doi: 10.1007/s00330-023-09873-w (PMC10667410; doi:10.1007/s00330-023-09873-w)
Supplement: Supplementary file 1 — Supplementary file1 (PDF 355 KB) [file 330_2023_9873_MOESM1_ESM.pdf]

Supplemental Table 1. Parameters of MRI in two hospitals

	Hospital 1	Hospital 2
MRI machines	3T<br>(Discovery 750; GE Healthcare)	3T<br>(Discovery 750W; GE Healthcare)
T2-weighted image		
Sequence	Fat-saturated FSE<br>(T2 Propeller)	Fat-saturated FSE<br>(T2 Propeller)
Repetition time/echo time (ms)	10000-13000/80-88	6300-8000/90-96
Parallel imaging factor	2.0	3.0
Matrix	320 × 320	320 × 320
Field of view (cm)	38 × 38	36 × 36
Section thickness/intersection gap (mm)	6/7	6/8
Number of excitations	2	2
Flip angle [degree]	110	142
T1-weighted gradient-echo image		
Sequence	3D T1 Dual Echo	3D T1 Dual Echo
Repetition time/echo time [ms]	3.9/2.3	5.3/1.3
Parallel imaging factor	2	2
Matrix	320× 200	256× 192
Field of view [cm]	38 × 38	38×38
Section thickness/intersection gap [mm]	4/2	5/2.5
Number of excitations	1	1
Flip angle [degree]	12	15
Diffusion-weighted image		
Sequence	Single-shot SE-EPI	Single-shot SE-EPI
Repetition time/echo time [ms]	6000-8000/52	16000/67
Parallel imaging factor	2	2
Matrix	128 × 160	128 × 98
Field of view [cm]	36 × 36	36 × 36
Section thickness/intersection gap [mm]	6/7	6/8
Number of excitations	8	8
Flip angle [degree]	90	90
b-value [s/mm2]	0, 800	0,1000
Motion-proving gradients	3 axes (x (RL), y (AP), and z (SI))	3 axes (x (RL), y (AP), and z (SI))
T1-wighted gradient-echo image		
Sequence	3D-GRE T1WI (LAVA)	3D-GRE T1WI (LAVA)
Repetition time/echo time [ms]	4.1/1.9	3.9/1.4
Parallel imaging factor	2	2
Matrix	320× 200	256 × 224
Field of view [cm]	38 × 38	40 × 40
Section thickness/intersection gap	4/2	4.4/2.2

[mm]		
Number of excitations	1	1
Flip angle [degree]	12	12
Scan delay after injection	Pre-contrast, 20 – 30 s, 45-52s, 75-82s, 135-142s	Pre-contrast, 20 – 30 s, 49-55s, 76-90s, 140-150s

Abbreviations: *MRI* magnetic resonance imaging, *FSE* fast spin echo, *TSE* turbo spin echo, *2D* 2-dimensional, *3D* 3-dimensional, *GRE* gradient echo, *SE* spin echo, *EPI* echo-planar imaging, *RL* right-left, *AP* anterior-posterior, *SI* superior-inferior, *T1WI* T1-weighted imaging, *T2WI* T2-weighted imaging, *LAVA* liver acquisition with volume acceleration.

Supplemental Table 2. The definition of qualitative imaging parameters

	Definition
Location	i) Left: S2-S4 according to Couinaud classification<br>ii) Right: S5-S8 according to Couinaud classification<br>iii) Caudate lobe
shape	i) Round or oval: round or oval shape<br>ii) Lobulated：an appearance resembling lobules<br>iii) Irregular: others
Contour	i) Smooth: the outline of the tumor not jagged or sharply angled<br>ii) Non-smooth: others
T2WI	i) Homogeneous: uniform signal<br>ii) Heterogeneous: mix signal
DWI	i) Homogeneous: uniform signal<br>ii) Heterogeneous: mix signal
Blood products	A nonenhancing defect with heterogeneous signal and amorphous or geographic in shape. The signal characteristics that depend on their acuity:<br>• Acute (hours to days): T1 hypo or iso, T2 hypo<br>• Subacute (days to months): T1 hyper, T2 variable<br>• Chronic (months to years): T1 hypo, T2 hypo.
Necrosis	A persistent, nonenhancing defect with either high signal intensity or low signal intensity (coagulation necrosis) on the T2WI
Upper abdominal lymphadenopathy	Lymph nodes > 8 mm on the short axis
Peritumoral bile duct dilatation	Bile duct dilation peripheral to tumour
Hepatic capsular retraction	Liver surface contour retraction
Cirrhosis	A lobulated/nodular contour and/or volume redistribution to the left lobe and caudate
Dynamic enhancement pattern	i) Progression: a continuous increase in signal intensity throughout time<br>ii) Fast-in and fast-out: an initial increase in signal intensity at arterial phase and subsequent decrease in signal intensity at postarterial phase<br>iii) Fast-in and slow-out: an increase in signal intensity at arterial phase and decrease in signal intensity at equilibrium phase or delayed phase, while signal intensity at portal venous phase can be the same as or slightly higher than the signal intensity at arterial phase<br>iv) Others: patterns that not mentioned above
Enhancement type	i) Hypoenhancing: nonhyperenhancing enhancement type<br>ii) Hyperenhancing: any part of the lesion showing higher signal than that of liver parenchyma in arterial phase
Degree of arterial phase enhancement	i) None: no arterial enhancement<br>ii) Mild-moderate: the enhancement being less than the aorta<br>iii) Strong: any part of the lesion showing similar enhancement to the aorta
Arterial phase enhancement pattern	i) Rim enhancement: ring-like enhancement with relatively hypoenhancing central areas at arterial phase<br>ii) Overall enhancement: enhancement in > 70% of the tumor area at arterial

	phase<br>iii) Partial enhancement: others
Peripheral washout at portal venous phase	A signal intensity reduction mainly in the peripheral part of the lesion at portal venous phase
Rim enhancement at portal venous phase	Ring-like enhancement with relatively hypoenhancing central areas at portal venous phase
Dot- or band-like enhancement inside the tumor	Presence of an area of nodular or thick line-shaped internal mass-enhancement in any phase of enhancement
Peripheral hepatic enhancement	Detectable relatively high signal regions in the liver parenchyma adjacent to or surrounding the lesion
Vessel penetrating the tumor	The presence of penetration vessels (hepatic artery, portal vein, or hepatic vein) in the lesion
Vessel encasement	Tumor involvement with the vessel surface or that produced vascular deformity
Portal venous thrombosis	Any persistent, nonenhancing defect in the portal venous

Supplemental Table 3. Comparison of the quantitative imaging parameters between IMCC and solitary CRLM in the training cohort

	Patients with IMCC (n=122)	Patients with CRLM (n=141)	*P* value
Thickness of arterial phase rim enhancement (mm)	10.6 (7.2)	8.6 (5.4)	0.055
Maximal diameter (mm)	63.0 (27.6)	36.0 (24.6)	<0.001*
LLC at precontrast phase	-0.14 (0.15)	-0.17 (0.19)	0.271
LLC at arterial phase	-0.04 (0.26)	-0.05 (0.25)	0.703
LLC at portal venous phase	-0.13 (0.30)	-0.20 (0.34)	0.731
LLC at delayed phase	-0.08 (0.50)	-0.17 (0.32)	0.742

Abbreviations: *IMCC* intrahepatic mass-forming cholangiocarcinoma, *CRLM* colorectal liver metastasis, *LLC* Lesion-to-liver contrast.

**P* value < .05.

Supplemental Table 4. Interobserver agreement for qualitative MRI findings

	Kappa value
Location	1.000
shape	0.923
Contour	0.632
T2WI	0.741
DWI	0.856
Blood products	0.948
Necrosis	0.800
Upper abdominal lymphadenopathy	0.912
Peritumoral bile duct dilatation	0.962
Hepatic capsular retraction	0.925
Cirrhosis	0.992
Dynamic enhancement pattern	0.821
Enhancement type	0.859
Degree of arterial phase enhancement	0.863
Arterial phase enhancement pattern	0.789
Peripheral washout at portal venous phase	0.767
Rim enhancement at portal venous phase	0.879
Dot- or band-like enhancement inside the tumor	0.706
Peripheral hepatic enhancement	0.933
Vessel penetrating the tumor	0.818
Vessel encasement	0.885
Portal venous thrombosis	0.963

Supplemental Table 5. Interobserver agreement for quantitative MRI findings

	Intraclass correlation coefficient
Thickness of arterial phase rim enhancement	0.822
Maximal diameter	0.966
LLC at precontrast phase	0.818
LLC at arterial phase	0.854
LLC at portal venous phase	0.884
LLC at delayed phase	0.804

Supplemental Table 6. Ridge regression results of MRI features (k = 0.60)

	Unstandardized Coefficients		t	*p*	R^2	*P*
	B	S.E.				
Constant	1.038	0.088	11.812	<0.001*		
T2WI	-0.041	0.024	-1.748	0.082		
Position	0.046	0.024	1.917	0.056		
MaxDiameter	-0.001	0.000	-1.912	0.057		
Shape	-0.048	0.012	-3.851	<0.001*		
Contour	0.051	0.023	2.161	0.032		
Cirrhosis	-0.129	0.043	-3.044	0.003*		
Peritumoral bile duct dilation	-0.055	0.029	-1.906	0.058		
Hepatic capsular retraction	-0.136	0.025	-5.388	<0.001*		
DWI	-0.043	0.028	-1.576	0.116		
Upper abdominal lymphadenopathy	-0.146	0.026	-5.712	<0.001*		
Enhancement type	-0.110	0.025	-4.391	<0.001*	0.649	<0.001*
Dynamic enhancement pattern	-0.036	0.011	-3.273	0.001*		
Arterial phase enhancement pattern	-0.020	0.014	-1.356	0.177		
Peripheral washout at portal venous phase	0.105	0.030	3.519	0.001*		
Rim enhancement at portal venous phase	0.109	0.030	3.570	<0.001*		
Peripheral hepatic enhancement	-0.158	0.025	-6.261	<0.001*		
Dot- or band-like enhancement inside the tumor	0.023	0.025	0.948	0.344		
Portal venous thrombosis	-0.034	0.038	-0.880	0.379		
Vessel penetrating the tumor	-0.154	0.024	-6.393	<0.001*		

Abbreviations: *T2WI* T2-weighted imaging, *DWI* diffusion-weighted imaging.

**p* value < .05.

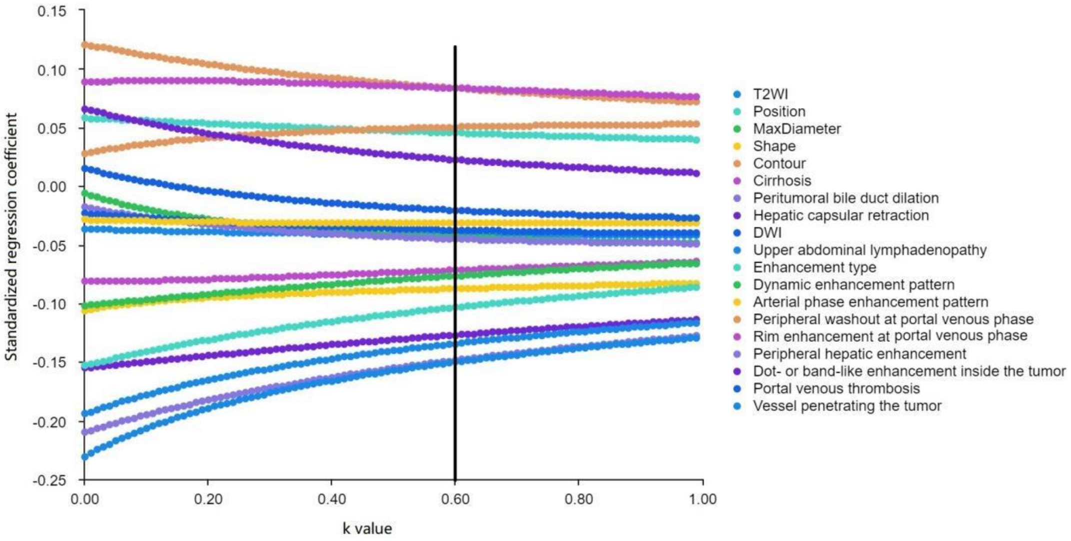

Supplemental Figure 1. The ridge curve of the relevant predictors for distinguishing IMCC from solitary CRLM
